# Classification of Dysphonic Voices in Parkinson’s Disease with Semi-Supervised Competitive Learning Algorithm

**DOI:** 10.3390/bios12070502

**Published:** 2022-07-09

**Authors:** Guidong Bao, Mengchen Lin, Xiaoqian Sang, Yangcan Hou, Yixuan Liu, Yunfeng Wu

**Affiliations:** School of Informatics, Xiamen University, 422 Si Ming South Road, Xiamen 361005, China; 23320201153974@stu.xmu.edu.cn (G.B.); 23320201154003@stu.xmu.edu.cn (M.L.); 23320201154016@stu.xmu.edu.cn (X.S.); 23320191153267@stu.xmu.edu.cn (Y.H.); 23320181154322@stu.xmu.edu.cn (Y.L.)

**Keywords:** Parkinson’s disease, semi-supervised learning, dysphonia, K-means clustering, competitive learning, k-nearest neighbor, pattern recognition

## Abstract

This article proposes a novel semi-supervised competitive learning (SSCL) algorithm for vocal pattern classifications in Parkinson’s disease (PD). The acoustic parameters of voice records were grouped into the families of jitter, shimmer, harmonic-to-noise, frequency, and nonlinear measures, respectively. The linear correlations were computed within each acoustic parameter family. According to the correlation matrix results, the jitter, shimmer, and harmonic-to-noise parameters presented as highly correlated in terms of Pearson’s correlation coefficients. Then, the principal component analysis (PCA) technique was implemented to eliminate the redundant dimensions of the acoustic parameters for each family. The Mann–Whitney–Wilcoxon hypothesis test was used to evaluate the significant difference of the PCA-projected features between the healthy subjects and PD patients. Eight dominant PCA-projected features were selected based on the eigenvalue threshold criterion and the statistical significance level (*p* < 0.05) of the hypothesis test. The SSCL algorithm proposed in this paper included the procedures of the competitive prototype seed selection, K-means optimization, and the nearest neighbor classifications. The pattern classification experimental results showed that the proposed SSCL method can provide the excellent diagnostic performances in terms of accuracy (0.838), recall (0.825), specificity (0.85), precision (0.846), F-score (0.835), Matthews correlation coefficient (0.675), area under the receiver operating characteristic curve (0.939), and Kappa coefficient (0.675), which were consistently better than those results of conventional KNN or SVM classifiers.

## 1. Introduction

Parkinson’s disease (PD) is a type of chronic neurological disorder that is progressively caused by the deterioration of neurotransmitter dopaminergic nerve cells in the substantia nigra of the brain [1]. In the early stages, PD patients occasionally suffer noticeable tremors in just one hand. Later on, PD patients gradually start experiencing some behavioral changes, with the primary symptoms including recurring tremors, stiffness, impaired balance, slow movement, freezing of gait, and dysphonia [2,3,4]. The dysfunction of body motor coordination in PD may also lead to several mental disturbances, with the secondary symptoms such as anxiety, depression, or fatigue. The development of muscle rigidity and articulatory hypokinesia in PD may cause some phonatory disorders such as dysphonia and hypokinetic dysarthria [5,6].

As a result of glottic insufficiency and vocal cord dysfunction in abduction or adduction, the altered acoustic amplitude and pitch frequency variations are commonly present in the speeches of PD patients [7,8]. In clinical practice, the changes of perceptual system and phonation impairment can be observed when PD patients pronounce sustained vowel sounds [5]. As an alternative clinical approach to medical imaging examination, the standard speech test is a low-cost and objective examination and monitoring solution, without any external radiation exposure from medical devices [9].

Voice records are often quantified in terms of various vocal parameters [10,11], which can be used as dominant biomarkers to detect the progressive changes of PD symptoms [12,13,14]. The acoustic signal amplitude (shimmer) parameters [15], pitch local perturbation (jitter) parameters [16], frequency cepstral parameters [17], vocal fold vibration periodicity (harmonics-to-noise ratio) parameters [18], and nonlinear dynamics measures [19], are the typical vocal parameters for quantifying the pathological conditions of dysphonia and dysarthria in PD [20,21]. The work of Rahn et al. [16] indicated that the jitter would significantly increase in PD patients. Little et al. [19] computed the correlation dimension and detrended fluctuation analysis (DFA) nonlinear features, and reported that the level of acoustic dynamics in the voice recordings of PD patients was much higher than that of healthy controls (HC). Cnockaert et al. [22] used the continuous wavelet transforms to study the fundamental frequency trace and low-frequency vocal modulation in sustained vowel voices, and they observed the significant phonatory disturbances in fundamental frequency of PD patients. Rodriguez-Perez et al. [23] reported that the mean, minimum, maximum, and standard deviation parameters of fundamental frequency modulation spectrum were significantly altered in PD. Viswanathan et al. [24] used the fractal dimension and normalized mutual information to quantify the complexity of acoustic signals in PD. They observed that the vocal fractal dimension of PD patients was significantly lower, and the normalized mutual information of sustained vowel voices in PD was significantly larger than in HC subjects [24]. They also compared the other nonlinear features such as normalized pitch period entropy and glottal closing quotient, and carried out the classification experiments using a support vector machine (SVM) based on a linear kernel. Their experimental results indicated that the fractal dimension and normalized mutual information were the dominant nonlinear features for detection of PD voices [24].

As some acoustic features are quantified using the similar signal processing tools, feature correlation analysis and selection procedures could be considered to reduce the feature dimensions and prevent the unnecessary computation costs [25,26]. Mohamadzadeh et al. [26] utilized a sparse representation technique to select the distinct features, and then categorized the vocal patterns of PD patients using an approximate message passing classifier. The vocal features may also help develop different effective computer-aided diagnostic tools that are capable of providing high sensitivity and specificity results for the detection of the symptomatic speech changes of PD patients [25,27].

Recently, advanced machine learning algorithms have been widely used to detection of vocal patterns for monitoring the PD progression and assessing the disease severity [20,28,29,30,31]. How to design a sensitively and reliably machine learning system for phonation impairment detection is still an engineering challenge. Vaiciukynas et al. [32] used the random forest algorithm to analyze the abnormal sustained vowel voices of PD patients. They reported that the nonlinear projection of a proximity matrix into the two-dimensional feature space can provide excellent feature visualizations to support the medical decision-making. Berus et al. [29] studied the vocal feature correlations using different correlation coefficients, and implemented dimensionality reduction using principal component analysis (PCA) and self-organizing maps. They also developed a number of feedforward artificial neural networks with different parameter configurations, and achieved an accuracy of 0.8647 in the voice classification tasks. Hires et al. [33] developed a hybrid system that combined multiple convolutional neural networks (CNNs) to distinguish the vocal patterns of PD patients. In order to train each CNN, they applied a multiple-fine-tuning approach to reduce the semantical gap between the source and target tasks. They reported that such a CNN ensemble system did not require any feature extraction procedure, and can also achieve excellent diagnostic results [33]. Sheibani et al. [34] designed a classifier ensemble system to identify the pathological vocal patterns with frequency features. Their experiments demonstrated that the ensemble system may provide an accuracy of 0.906, which was better than a single classifier. Machine learning algorithms may assist in designing portable computer-aided systems for speech monitoring as well. Lauraitis et al. [35] developed a mobile application with Android operating system to record the body movement and speech data from patients with neurological disorders and HC subjects. Their experiments indicated that the finger tapping, energy expenditure, and vocal features were useful for different neural impairment monitoring.

In this paper, we propose a novel semi-supervised learning method based on competitive learning to detect the dysphonic voice recordings of PD patients. The acoustic parameters extracted from 240 voice records of PD patients and age-matched HC subjects were analyzed by grouping into the families of jitter, shimmer, harmonic-to-noise ratio, frequency cepstral parameters, and nonlinear measures. The Pearson correlation analysis and the PCA method were applied to remove the linear correlations between vocal parameters and reduce the feature dimensions, respectively. The dominant PCA-projected features were selected based on the Mann–Whitney–Wilcoxon hypothesis test (*p*-values < 0.05) for further pattern classification tasks. The semi-supervised competitive learning method for detection of vocal patterns in PD contains the procedures of the competitive prototype seed selection, K-means optimization, and the nearest neighbor classifiers. The effectiveness of the proposed machine learning method was evaluated in terms of confusion matrix and several prevailing diagnostic metrics.

## 2. Materials

The set of dysphonic voice data named “Parkinson Dataset with replicated acoustic features Data Set” was provided by Naranjo et al. [36], which is publicly available via University of California, Irvine (UCI) machine learning repository [37]. As reported by Naranjo et al. [36], a total of 80 subjects participated in the voice recording experiment. The number of HC subjects (n=40) matched that of PD patients (n=40). The HC subject group consisted of 22 (55%) healthy males and 18 (45%) healthy females, with the averaged age of 66.38 ± 8.38 years old. The age-matched PD group (mean ± SD: 69.58 ± 7.82 years old) was composed of 27 (67.5%) male and 13 (32.5%) female patients, respectively, who were recruited from the Regional Association for Parkinson’s Disease in Extremadura, Spain. At least two of the primary symptoms of resting tremor at 4-6 Hz, muscle rigidity, postural instability, or bradykinesia were manifested in these PD patients. All these subjects provided their signed informed consent sheets, by complying with the protocol of the voice recording experiments [36]. The protocol documents were reviewed and approved by the Bioethical Committee from the University of Extremadura, Spain, as claimed in the previous study of Naranjo et al. [36]. The population statistics of HC subjects and PD patients are listed in Table 1.

During the voice recording process, the subjects pronounced the sustained vowel sounds at their normal speed for 5 s. Each subject was requested to repeat the vowel sounds for three times, so that three separate voices were recorded from one subject. Thus, the dysphonic voice data set contains 240 voice records in total. According to Naranjo et al. [36], the voices were acquired by a microphone (AKG Model: 520) and recorded by a laptop with an external sound card (TASCAM Model: US322). The raw voice recordings were sampled at 44.1 kHz and digitalized with a resolution of 16 bits per sample [36].

The acoustic parameters derived from the voice recordings were grouped into the following five families: pitch local perturbation (jitter) measures, amplitude local perturbation (shimmer) measures, harmonic-to-noise (HNR) features, nonlinear measures, Mel-frequency cepstral coefficient (MFCC), and frequency delta measures [17,36]. The nonlinear parameters were computed using the prevailing measures such as recurrence period density entropy (RPDE), DFA, pitch period entropy (PPE), and glottal-to-noise excitation ratio (GNE), respectively. Table 2 lists the abbreviations of the acoustic parameters and the detailed descriptions for the jitter, shimmer, HNR, nonlinear, and frequency MFCC and Delta families, respectively.

## 3. Methods

**Figure 1:**
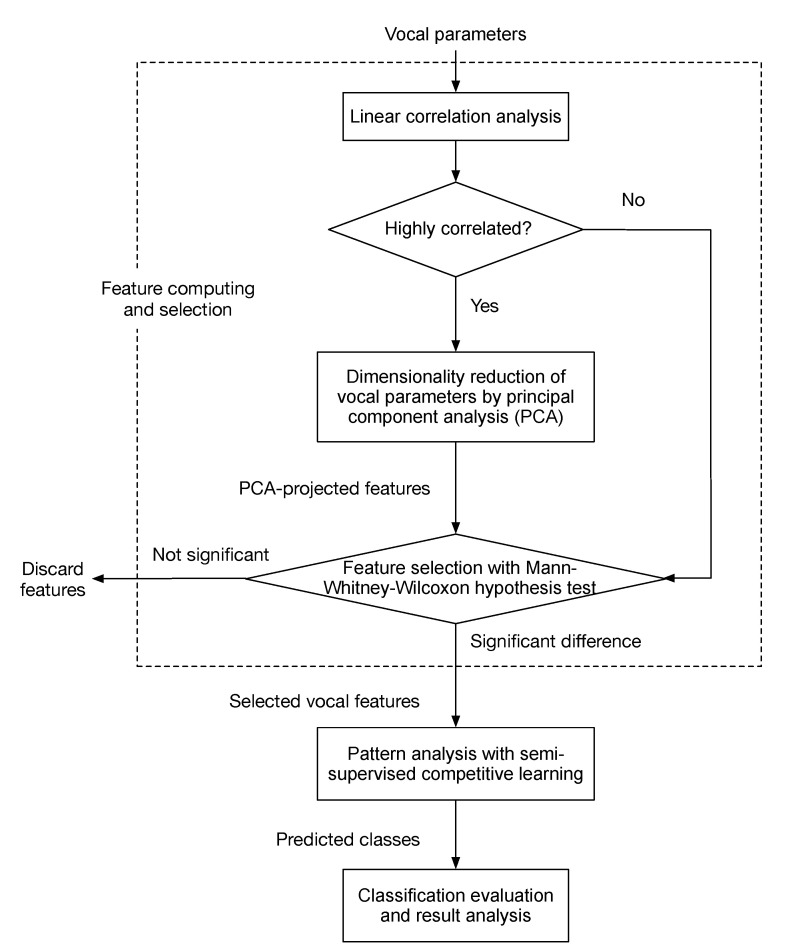
Flowchart of the voice detection procedures that contain vocal parameter analysis, dimensionality reduction, feature selection using the Mann–Whitney–Wilcoxon hypothesis test, pattern analysis based on semi-supervised competitive learning, and classification result evaluation.

The present study developed the methodological procedures of feature computing with dimensionality reduction, pattern analysis based on semi-supervised learning, classification performance evaluation and result analysis [38]. The flowchart of the proposed method is shown in Figure 1.

### 3.1. Feature Computing and Selection

According to the previous work of Naranjo et al. [17], the vocal parameters computed from the voice recordings of each subject in the data set would manifest high correlations between each other. In the present study, we grouped the vocal parameters into five families, i.e., the jitter, shimmer, harmonic-to-noise, nonlinear, and frequency parameter families. Then, we studied the linear correlations of the vocal parameters within each family by calculating the Pearson correlation coefficients as

(1) $$\rho_{a,b}=\frac{cov\left(V_{a},V_{b}\right)}{SD_{a}\cdot SD_{b}},$$

where $\mathrm{cov}\left(V_{a},V_{b}\right)$ is the covariance of a pair of vocal parameters $V_{a}$ and $V_{b}$, and SD denotes the standard deviation.

Regarding those vocal parameter families that presented strong linear correlations ($\left|\rho \right|>0.75$), it is necessary to decrease the correlated redundancy [39]. In the present study, we applied the PCA method to reduce the dimensions in feature space. The PCA is an orthogonal linear transformation that derives the eigenvalues and the corresponding eigenvectors from the covariance matrix of the data. The components can be projected based on the weight matrix in accordance with the eigenvalues sorted from largest to smallest. The dimension reduction procedure was implemented according to the 90% percentage threshold criterion, i.e., the first *h* principal components would be kept if the sum of their eigenvalues over the sum of total eigenvalues reached a certain percentage of 90% as:

(2) $$h=\arg\min_{h}\left\{\frac{\sum_{q=1}^{h}\lambda_{V_{q}}}{\sum_{q=1}^{Q}\lambda_{V_{q}}}\geq 90\%\right\},\;h\leq Q,$$

where *Q* denotes the total number of eigenvalues $\lambda_{V_{q}}$.

The selection of the PCA-projected acoustic parameters, along with the remaining weakly correlated parameters, was performed using statistical analysis of the Mann–Whitney–Wilcoxon hypothesis test, which may estimate whether the statistic value from two populations was equal without a strict normal distribution assumption. The level of statistical significance was set as *p*-value < 0.05. Only the vocal parameters that presented the significant difference would be selected to construct the vector of input features for further pattern analysis.

### 3.2. Pattern Analysis with Semi-Supervised Competitive Learning

#### 3.2.1. Competitive Selection of Initial Prototype Seeds

Given a *L*-dimensional data set of size *N*, expressed as the set $X=\{x_{1},x_{2},\cdots ,x_{N}\}$, let us define the L2 norm to measure the Euclidean distance between a pair of data patterns in the *L* feature dimensions as

(3) $$dist\left(x_{m},x_{n}\right)={∥x_{m}-x_{n}∥}_{2}=\sqrt{\sum_{l=1}^{L}{\left(x_{m}^{l}-x_{n}^{l}\right)}^{2}}.$$

The indicator function of $I\left(x_{m},x_{n},\eta \right)$ is defined to represent the proximity degree between two data patterns as follows

(4) $$I\left(x_{m},x_{n},\eta \right)=\left\{\begin{array}{ll} 1, & \mathrm{if}\;dist\left(x_{m},x_{n}\right)\leq \eta ,\\ 0, & \mathrm{otherwise}. \end{array}\right.$$

If the Euclidean distance between $x_{m}$ and $x_{n}$ is not larger than an assigned range η, the indicator function equals 1, otherwise the indicator value remains zero. Then, the density of $x_{m}$ can be calculated as the sum of its indicator values with regards to all of the patterns in the data set, i.e.,

(5) $$D\left(x_{m}\right)=\sum_{n=1}^{N}I(x_{m},x_{n},\eta )$$

A candidate seed, $x_{m}$, is selected according to the following indicator function:

(6) $$C\left(x_{m},\gamma \right)=\left\{\begin{array}{cc} 1, & \mathrm{if}\;D(x_{m})\geq \gamma ,\\ 0, & \mathrm{otherwise}, \end{array}\right.$$

where γ (1<γ≤N) is the density threshold parameter. The set of all available candidate seeds, C (C⊆X), is composed of the data patterns whose densities reach over the threshold as

(7) $$C=\left\{x_{m}\left|\;C(x_{m},\gamma )=1,\right.\;\forall x_{m}\in X\right\}.$$

Let us define $A(x_{m})$ to be the set of all adjacent candidate seeds that locate in the range η of the candidate seed $C(x_{m})$, i.e.,

(8) $$A\left(x_{m}\right)=\left\{x_{n}\left|\;I\left(x_{m},x_{n},\eta \right)\cdot C\left(x_{m},\gamma \right)=1,\right.\;\forall x_{n}\in X\right\}.$$

It is worth noting that the candidate seed $x_{m}$ itself also belongs to the adjacent set $A(x_{m})$, and $A(x_{m})$ is a subset of the entire data set X, i.e., $x_{m}\in A\left(x_{m}\right)$ and $A(x_{m})\subset X$.

#### 3.2.2. K-Means Optimization Algorithm

The qualified prototype seeds can be selected during the competitive learning process based on the *K*-means optimization algorithm. For a data pattern $x_{n}$ that belongs to the prototype *k*, its prototype indicator parameter can be written as

(9) $$\theta_{kn}=\left\{\begin{array}{ll} 1 & \mathrm{if}\;x_{n}\in \mathrm{prototype}\;k,\\ 0 & \mathrm{otherwise}. \end{array}\right.$$

The effectiveness and sensitivity of the *K*-means learning algorithm mainly depend on the choice of the number of prototypes *K* and the initialization of the centroid seeds, respectively. Let S (S⊂X) denote the set of winning prototype seeds. In the *K*-means procedure, the number of total prototypes is determined by the size of the set of prototype seeds S, instead of arbitrary assignment. Additionally, the members of S obtained by the previous competitive selection process are used as the initial centroids of the prototypes for *K*-means optimization, i.e., $\left\{s_{k}^{0}\right\}=S^{*}$, because the data patterns with the relatively higher densities are most likely close to the true prototype centroids.

The *K*-means learning algorithm iteratively searches for the optimal partitions of data patterns by following the minimum sum of squared error criterion, and then updates the prototype centroids. In the *i*th *K*-means optimization iteration, the sum of squared errors between the data patterns $x_{n}$ and the prototype centroids $s_{k}^{i}$ is optimized with respect to the indicators $\theta_{kn}^{i}$ as

(10) $$\left\{\begin{array}{c} \min\sum_{k=1}^{K}\sum_{n=1}^{N}\theta_{kn}^{i}{∥x_{n}-s_{k}^{i}∥}_{2},\\ s.t.\;\sum_{k=1}^{K}\theta_{kn}^{i}=1,\;\forall x_{n}\in X. \end{array}\right.$$

Then, the prototype centroids $s_{k}^{i+1}$ are updated for the i+1th iteration as

(11) $$s_{k}^{i+1}=\frac{\sum_{n=1}^{N}\theta_{kn}^{i}x_{n}}{\sum_{n=1}^{N}\theta_{kn}^{i}}$$

Such an optimization iteration and the corresponding centroid update are repeated until the location for each prototype centroid no longer changes.

#### 3.2.3. Nearest Neighbor Classification

In the present study, we utilized the concept of nearest neighbor clustering to accomplish the pattern analysis tasks. The nearest neighbor algorithm aims to categorize the data patterns that belong to the prototype *k* into the target class labels based on the *P*-size training set, $P={\left\{x_{p},\omega_{p}\right\}}_{p=1}^{P}$. Such a training set contains the available data patterns $x_{p}$ with known labels $\omega_{p}$ for the training purpose. In the case that the amount of training data (the patterns with known class labels) are less that the total number of testing data (the prototype patterns to be distinguished), i.e., P<N, we may use each prototype centroid as a representative of its own prototype patterns.

Let ${\left\{x_{kp},\omega_{kp}\right\}}_{p=1}^{R}$ denote the *R* (R≤P) nearest neighbors of a query prototype centroid $s_{k}$, in terms of the first *R* smallest Euclidean distance between the training patterns and the prototype centroid $s_{k}$, i.e.,

(12) $${\left\{x_{kp},\omega_{kp}\right\}}_{p=1}^{R}=\underset{\{x_{p},\omega_{p}\}\;\subseteq \;P}{\arg\mathrm{sort}}dist\left(s_{k},x_{p}\right).$$

Let $\tilde{\omega }={\left\{\omega^{t}\right\}}_{t=1}^{T}$ represent the set of target class labels, where T(T≤P) is the total number of target classes. We may define the class indicator function $\delta \left(\omega_{kp},\omega^{t}\right)$ to indicate the circumstance if $\omega_{kp}$ matches $\omega^{t}$ as

(13) $$\delta \left(\omega_{kp},\omega^{t}\right)=\left\{\begin{array}{ll} 1, & \mathrm{if}\;\omega_{kp}=\omega^{t},\\ 0, & \mathrm{otherwise}. \end{array}\right.$$

Then, the class label $\omega_{s_{k}}$ of the query prototype centroid $s_{k}$ is predicted by the majority vote of the labels of *R* nearest neighbors as

(14) $$\omega_{s_{k}}=\underset{\omega^{t}\in \tilde{\omega }}{\arg\max}\sum_{p=1}^{R}\delta \left(\omega_{kp},\omega^{t}\right).$$

Finally, all of the data patterns that belong to the prototype *k* are assigned with the same class label as the centroid $s_{k}$ as the overall classification results of our semi-supervised competitive learning method, i.e.,

(15) $$\omega_{n}\left(x_{n}\in \mathrm{prototype}\;k\right)=\omega_{s_{k}}.$$

### 3.3. Benchmark Classifiers for Comparison

In order to compare the classification results, we also applied the benchmark classifiers, in particular *K*-nearest neighbor (KNN) and SVM, on the same dysphonic voice data set. The parameter K=7 was selected as the number of nearest neighbor patterns with known class labels, with the aim to best predict the classes of the testing patterns by means of majority voting.

The SVM is a typical feedforward neural network that consists of a single nonlinear hidden layer. Unlike the multilayer perceptron trained by the well-known back-propagation algorithm, the SVM operates only in a batch mode and follows the principle of structural risk minimization, which roots in the Vapnik–Chervonenkis dimension theory [40]. The SVM training is achieved by optimizing the margin hyperplane that separates the training data samples with several slack variables (also referred to as support vectors). For the nonlinear classification problems, the data samples are commonly projected into a high-dimensional space by the inner-product kernel function such that the pattern classification can be converted into a linear separable problem.

In the present study, the SVM kernel was constructed with a radial basis function. The optimal spread parameter of the radial basis function was selected as σ=4, so as to provide the best classification accuracy. The training and testing of the KNN, SVM, and SSCL methods were implemented by following the 10-fold cross-validation evaluation approach. In addition, an expert system based on Bayesian inference [36] and a two-stage variable selection and classification method [17] were used for a comparison of previous related studies on the same data set.

### 3.4. Classification Performance Evaluation Metrics

In order to evaluate the voice detection and classification results, we considered the prevailing pattern analysis metrics such as accuracy, recall, specificity, precision, F-score, the Matthews correlation coefficient, the area under the receiver operating characteristic (ROC) curve [4], and Cohen’s Kappa coefficient.

#### 3.4.1. Accuracy

The confusion matrix was calculated with the ratios of true positive (TP), true negative (TN), false positive (FP, also referred to as Type I error), and false negative (FN, referred to as Type II error). The overall accuracy of an entire data classification can be written as

(16) $$\mathrm{Accuracy}=\frac{\mathrm{TP}+\mathrm{TN}}{\mathrm{TP}+\mathrm{TN}+\mathrm{FP}+\mathrm{FN}}.$$

#### 3.4.2. Recall

The recall, also referred to as sensitivity, describes the true positive rate of a given classification model, the definition of which is formulated as

(17) $$\mathrm{Recall}=\frac{\mathrm{TP}}{\mathrm{TP}+\mathrm{FN}}.$$

#### 3.4.3. Specificity

The specificity presents the true negative rate of a classification model, which is given by

(18) $$\mathrm{Specificity}=\frac{\mathrm{TN}}{\mathrm{FP}+\mathrm{TN}}.$$

#### 3.4.4. Precision

The precision is also referred to as positive predictive rate, i.e., the ratio of correct positive patterns to the total predicted positive patterns, defined by

(19) $$\mathrm{Precision}=\frac{\mathrm{TP}}{\mathrm{TP}+\mathrm{FP}}.$$

#### 3.4.5. F-Score

Because the numbers of HC and PD subjects and their voice records were matched in the UCI dysphonic voice data set, the F-score can be calculated as the harmonic mean of precision and recall based on the confusion matrix [41], derived as

(20) $$F\text{-}\mathrm{score}=2\frac{\mathrm{Precision}\times \mathrm{Recall}}{\mathrm{Precision}\;+\;\mathrm{Recall}}$$

#### 3.4.6. Matthews Correlation Coefficient

The Matthews correlation coefficient (MCC) [42] was commonly used as a classification quality indicator for performance evaluation, which can be calculated as

(21) $$\mathrm{MCC}=\frac{\mathrm{TP}\times \mathrm{TN}-\mathrm{FP}\times \mathrm{FN}}{\sqrt{\left(\mathrm{TP}+\mathrm{FP})\times (\mathrm{TP}+\mathrm{FN})\times (\mathrm{TN}+\mathrm{FP})\times (\mathrm{TN}+\mathrm{FN}\right)}}.$$

The typical random guess leads to a zero MCC value (MCC = 0). An effective binary classifier should provide a positive MCC value closer to perfect diagnosis (MCC = 1). The MCC is an important indicator because it considers all the true and false positives and negatives with the representation in the form of normalized correlation coefficient.

#### 3.4.7. Area under ROC Curve

The ROC curve is a graphical plot of diagnostic trade-off between the clinical recall/sensitivity and specificity for every possible cut-off for a combination of binary classification tests. The area under the ROC curve (AUC) estimates the area underneath the entire ROC curve, which is commonly used for the evaluation of the diagnostic performance in many biomedical applications. Typically, a classifier that generates a ROC curve closer to the top-left corner in the plot and with a larger AUC indicates an excellent diagnostic performance.

#### 3.4.8. Kappa Coefficient

In medical research, Cohen’s Kappa coefficient is a benchmark metric that evaluates the level of agreement between the predicted classes and the truth classes [43]. The Kappa coefficient attempts to measures the ratio of the observed agreement over the expected agreement purely by chance.

The calculation of Kappa coefficient can be expressed as [43]

(22) $$\mathrm{Kappa}=\frac{p_{o}-p_{e}}{1-p_{e}}.$$

The term $p_{o}$ is the observed agreement computed for the total number *n* of voice records as

(23) $$p_{o}=\frac{\mathrm{TP}+\mathrm{TN}}{n}.$$

The term $p_{e}$ is the hypothetical probability of expected agreement due to chance, defined as

(24) $$p_{e}=\frac{\left(\mathrm{TP}+\mathrm{FP})\times (\mathrm{TP}+\mathrm{FN})\times (\mathrm{TN}+\mathrm{FP})\times (\mathrm{TN}+\mathrm{FN}\right)}{n^{2}}.$$

The Kappa coefficient typically varies in the range from 0 and 1. A zero Kappa coefficient (Kappa = 0) indicates no agreement between the predicted classes and the truth classes, which can be interpreted to mean that the predictions made by a classifier are completely incorrect over the entire data set. A Kappa coefficient between 0.6 and 0.8 commonly implies a substantial agreement, and the Kappa coefficient over 0.8 toward 1 implies a near perfect agreement. In the present study, we computed the Kappa coefficient for each classification method to assess the classification performance.

## 4. Results and Discussions

### 4.1. Feature Analysis Results

**Figure 2:**
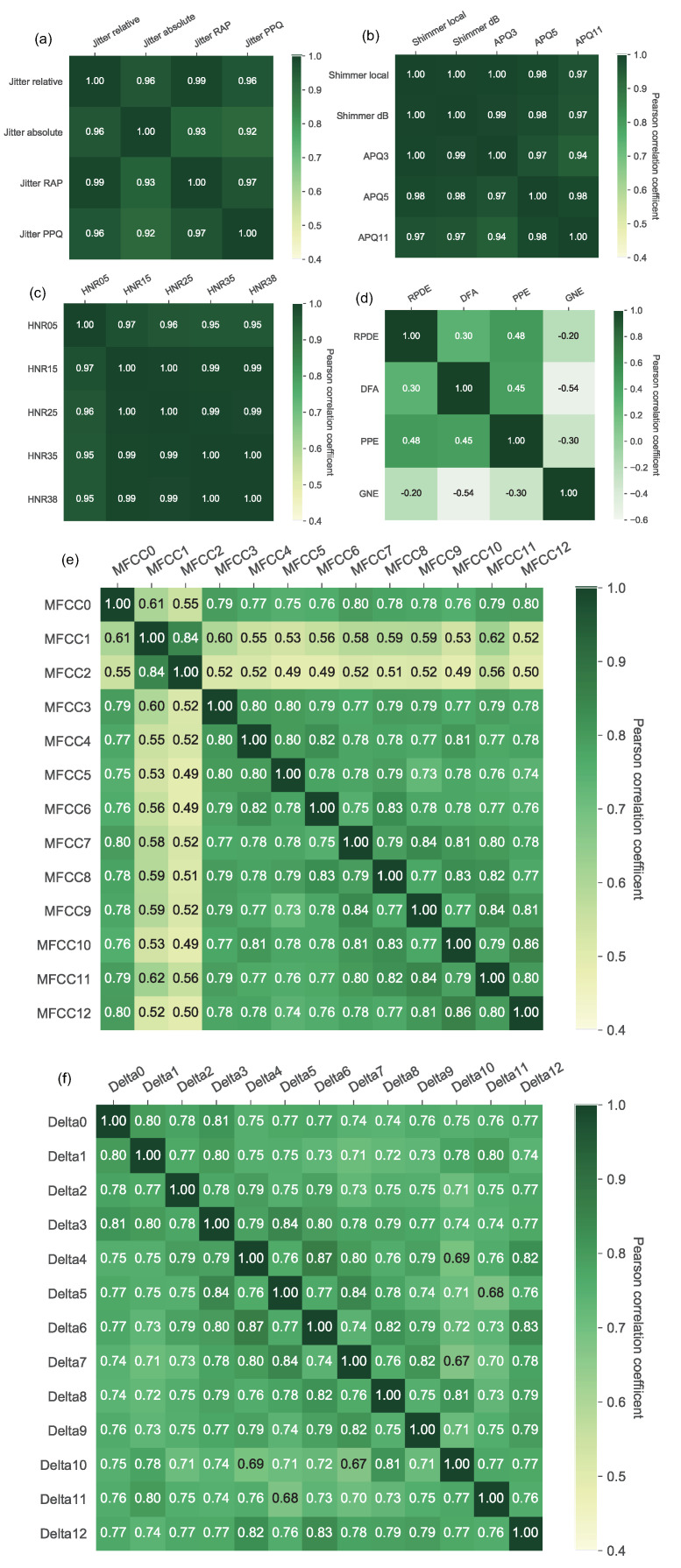
Pearson correlation coefficient results of the families of (**a**) Jitter, (**b**) Shimmer, (**c**) HNR, (**d**) Nonlinear, (**e**) MFCC, and (**f**) Frequency Delta vocal parameters.

Figure 2 provides the color maps of the Pearson correlation coefficients computed from each pair of vocal parameters for the families of jitter, shimmer, HNR, nonlinear, MFCC, and frequency delta, respectively. It is worth noting that the jitter, shimmer, and HNR vocal parameters were highly correlated (ρ≥0.92) between each other in each family. With regard to the MFCC and Delta families, the vocal parameters showed somewhat positive correlated but not strong enough (0<ρ<0.9). In contrast, the nonlinear vocal parameters of RPDE, DFA, PPE, and GNE only presented slight linear correlations with each other (−0.6<ρ<0.5). The similar acoustic signal preprocessing procedure for each family (listed in Table 2) was the major reason that caused the highly linear correlations of vocal parameters. On the other hand, the RPDE, DFA, PPE, and GNE parameters were computed by different nonlinear algorithms, so that these vocal parameters produced little correlated outcomes in the present study. In consequence, the RPDE, DFA, PPE, and GNE parameters were retained in the nonlinear parameter family, and the resting parameter families were projected by the PCA method into the principal component vectors in the lower dimensional feature space.

Because the jitter, shimmer, HNR, and frequency parameters manifested strong linear correlations as depicted in Figure 2, it is necessary to reduce the similarity effect of vocal parameters. By following the PCA dimension reduction criterion as described in Section 3.1, we only retained the primary principal components of the jitter, shimmer, and HNR parameter families for each, and annotated them as jitter-PCA, shimmer-PCA, and HNR-PCA, respectively. Regarding the frequency MFCC and Delta parameters, the first six and the first five principal components were chosen and annotated as Frequency-MFCC-PCA1 to 6 and Frequency-Delta-PCA1 to 5, respectively, as listed in Table 3.

The PCA-projected features does not imply that they are separable between two classes in statistical sense. Table 3 provides the statistical hypothesis test results and the *p*-values of the PCA-projected vocal features. The Mann–Whitney–Wilcoxon hypothesis test results indicated that the jitter-PCA, shimmer-PCA, and HNR-PCA consistently showed the significant difference (*p*-value < 0.05) between the HC and PD subject groups. Only the PPE and GNE nonlinear parameters of PD group were significantly different from those of HC group. However, the RPDE and DFA parameters of PD patients still maintained some degree of overlapping with those of HC subjects. Moreover, it is worth noting that the MFCC-PCA1, Delta-PCA1, and Delta-PCA5 parameters of the PD group were significantly different from those of the HC group, whereas the resting frequency parameters of MFCC and Delta families just showed slight differences that did not reach the statistical significance level of *p*-value < 0.05. Therefore, we selected the jitter-PCA, shimmer-PCA, HNR-PCA, PPE, GNE, MFCC-PCA1, Delta-PCA1, and Delta-PCA5 as the dominant features for pattern analysis based on the machine learning algorithms.

### 4.2. Classification Results and Discussions

Based on the selected eight dominant features, the pattern classification tasks were accomplished by the proposed SSCL method and the benchmark KNN and SVM classifiers. Table 4 demonstrated the classification results provided by different pattern analysis methods, along with the results reported in the previous studies [17,36] for comparison purpose. The best classification results were marked in bold numerical values in the table.

It is worth noting that most classification results such as accuracy, recall, specific, and precision reported by Narajo et al. [17,36] just ranged from 0.75 to 0.8. The results of the two-stage method were slightly better than those obtained with the Bayesian expert system. In the present study, the KNN (K=7), the SVM with radial basis function kernels, and the proposed SSCL method consistently outperformed the expert system with Bayesian inference, on the same data set. All of the KNN, SVM, and SSCL method provided higher values of accuracy, recall/sensitivity, and specificity, precision, and MCC, compared with the two-stage method used in [17]. In particular, the SVM ameliorated the classification performance with improvements of 0.046, 0.035, 0.058, 0.036 for accuracy, recall, specificity, and precision, respectively, versus the results of two-stage method. The increments of accuracy, recall, precision provided by the proposed SSCL method over the SVM classifier were 0.013, 0.025, and 0.004, respectively. Although the precision and AUC values of the KNN were a bit lower than those of the two-stage method, the KNN still provided a recall of 0.812 as true positive rate, which was much better than that of the Narajo’s two-stage method (recall: 0.765). Such classification results obtained with the benchmark KNN and SVM classifiers, along with the proposed SSCL method, demonstrated the merits of the correlation analysis and vocal feature dimension reduction procedures. In particular, the proposed SSCL method achieved the best in four confusion matrix metrics (accuracy: 0.838, recall: 0.825, specificity: 0.85, precision: 0.846), compared with the other benchmark classifiers or the latest research works [17,36]. It can be observed in Table 4 that the Kappa coefficients of the KNN, SVM, and SSCL methods were over 0.6, which indicated that three classifiers can provide the substantial agreements between the predicted classes and the truth classes. The SSCL method also provided the largest Kappa coefficient value (Kappa = 0.675), in comparison with either of the KNN or SVM classifier. It is also worth noting that the SD values of all classification metrics provided by the proposed SSCL method were consistently smaller than the other classifiers. Such results demonstrated that the SSCL method can effectively distinguish the overall vocal patterns between the HC and PD subject groups.

**Figure 3:**
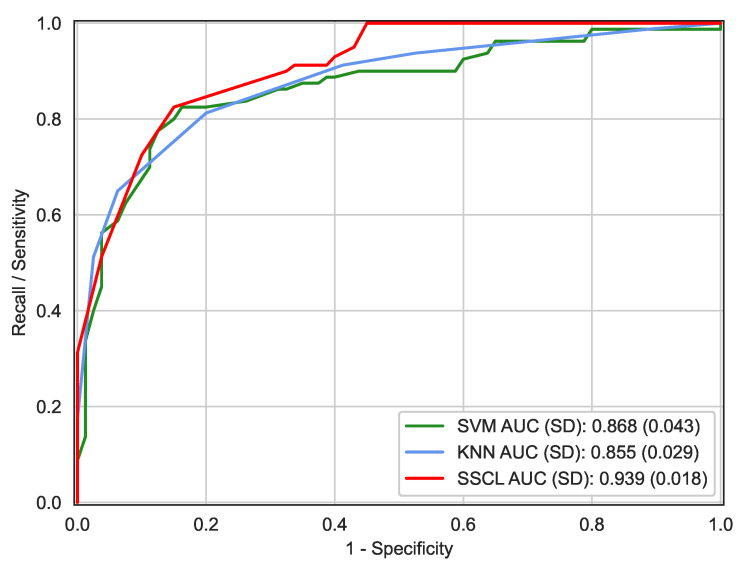
ROC curves generated by the SVM with radial basis functions, KNN, and SSCL methods, respectively. The AUC values estimated by the SVM, KNN, and SSCL methods were 0.868 ± 0.043, 0.855 ± 0.029, and 0.939 ± 0.018, respectively.

According to Figure 3, it can be observed that the ROC curve provided by the proposed SSCL method was consistently superior to the other two benchmark classifiers, especially when the false positive rate (i.e., 1−specificity) ranged from 0.1 to 1.0. Moreover, the proposed SSCL method improved by an AUC increase of 0.06 over the two-stage method proposed by Narajo et al. [17].

Our experimental results were also comparable to the other relevant research works in the literature. Viswanathan et al. [24] reported that the linear-kernel SVM input with the combination of fractal dimension and features extracted from three sustained voices provided the best classification accuracy of 0.81 and AUC value of 0.84, respectively. In our experiments, both of the SVM with radial basis function kernels and the proposed SSCL methods produced better overall accuracy results than the work of Viswanathan et al. [24]. Furthermore, the SVM and SSCL methods have greatly increased the AUC values up to 0.868 and 0.939, respectively.

Berus et al. [29] implemented the similar feature dimension reduction based on PCA and self-organizing maps, and used a group of artificial neural networks to distinguish vocal patterns between 20 HC subjects and 20 PD patients. Our proposed SSCL method provided a classification accuracy of 0.838, which is comparable to the accuracy result of 0.8647 reported by Berus et al. [29].

In addition, we studied the classification results of the KNN, SVM, and SSCL methods by considering the gender factor as well. Table 5 lists the misclassified voice records with regard to gender provided by the KNN, SVM, and SSCL methods, respectively. The misclassified voice recordings for males and females, as well as for HC and PD subject groups, were computed in percentage over the total incorrect voice recordings for each classifier. It is worth noting that all of the three classifiers tended to be good at distinguish vocal patterns in female, especially for PD patients. The misclassification ratios of males and females produced by both of the KNN and SVM were comparable for HC subjects. On the other hand, for PD patients, the misclassification ratios of females were much smaller than those of males. Such results were confirmed by the observation of Rusz et al. [44] that the speech abnormalities of females were generally better distinguished in relation to those of males.

## 5. Conclusions

The quality of phonation and daily communications of PD patients could be affected by the dysphonia, which can be detected based on the vocal parameters and machine learning algorithms. In the present study, the vocal patterns between HC subjects and PD patients have been effectively categorized using the selected PCA-projected features and the semi-supervised learning algorithm. The Pearson correlation coefficients were calculated between acoustic parameter pairs for the families of jitter, shimmer, HNR, nonlinear, and frequency MFCC and Delta, respectively. The experimental results showed strong linear correlations of jitter, shimmer, HNR parameters with each other in their own families. Validated by the Mann–Whitney–Wilcoxon hypothesis test, the PCA-projected features that presented significantly different in PD were selected for the purpose of dimension reduction. The semi-supervised machine learning method that incorporated the procedures of competitive prototype selection, K-means optimization, and the nearest neighbor classification was proposed for the pattern classification task. According to our classification experiments, the semi-supervised learning method was able to provide higher accuracy, recall, specificity, F-score, and Matthews correlation coefficient, and area under ROC curve, which was superior to the prevailing KNN and SVM classifiers. The proposed SSCL method also demonstrated better diagnostic performances than the results reported by Naranjo’s previous related studies.

It is believed that the feature selection and SSCL methods presented in this paper can also be used for classification of vocal patterns in other diseases. Because several vocal parameters are measured with similar settings, the feature selection method is able to reduce the correlated dimensions and avoid unnecessary computational costs. Then, the SSCL method can make effective classifications based on the dominant PCA-projected features. Validations of the feature selection and SSCL methods for analysis of the voice characteristics related to other diseases could be involved in the future work.

The future work could also focus on the nonlinear measure of voice disturbances associated with PD severity stages, and the advanced deep learning paradigms for the design of sensitively and reliably phonation impairment detection and monitoring systems.

## Figures and Tables

**Table 1:** Subject groups of HC and PD patients, with the age statistics presented as mean ± SD.

Subject Groups	HC Group		PD Group	
Gender	Male	Female	Male	Female
*n* (%)	22 (55%)	18 (45%)	27 (67.5%)	13 (32.5%)
Age (years old)	66.38 ± 8.38		69.58 ± 7.82	

**Table 2:** Description of acoustic parameter families derived from the voice records in HC and PD subject groups.

Parameter Family	Abbreviation	Parameter Description
Jitter	Jitter-Rel	Relative jitter
	Jitter-Abs	Absolute jitter
	Jitter-RAP	Relative average perturbation
	Jitter-PPQ	Pitch perturbation quotient
Shimmer	Shim-Loc	Local shimmer
	Shim-dB	Shimmer in dB
	Shim-APQ3	3-point amplitude perturbation quotient
	Shim-APQ5	5-point amplitude perturbation quotient
	Shim-APQ11	11-point amplitude perturbation quotient
Harmonic-to-noise	HNR05	Harmonic-to-noise ratio in 0–500 Hz
	HNR15	Harmonic-to-noise ratio in 0–1500 Hz
	HNR25	Harmonic-to-noise ratio in 0–2500 Hz
	HNR35	Harmonic-to-noise ratio in 0–3500 Hz
	HNR38	Harmonic-to-noise ratio in 0–3800 Hz
Nonlinear	RPDE	Recurrence period density entropy
	DFA	Detrended fluctuation analysis
	PPE	Pitch period entropy
	GNE	Glottal-to-noise excitation ratio
Frequency	MFCC 0 to 12	Mel-frequency cepstral coefficient-based spectral measures of order 0–12
	Delta 0 to 12	The derivatives of mel-frequency cepstral coefficient measures of order 0–12

**Table 3:** Mann–Whitney–Wilcoxon hypothesis test results of the vocal features derived from the PCA approach. The *p*-value < 0.05 indicates the significant difference, marked with *. Null hypothesis: Data samples from two subject groups are not significantly different in statistical sense; 1: rejects the null hypothesis, with the corresponding *p*-value marked with stars, 0: accepts the null hypothesis.

Vocal Features	Null Hypothesis	*p*-Value
Jitter-PCA	1	0.0036 *
Shimmer-PCA	1	0.0007 *
HNR-PCA	1	0.0001 *
Nonlinear-RPDE	0	0.1779
Nonlinear-DFA	0	0.3233
Nonlinear-PPE	1	0.0476 *
Nonlinear-GNE	1	0.0001 *
Frequency-MFCC-PCA1	1	0.0001 *
Frequency-MFCC-PCA2	0	0.2305
Frequency-MFCC-PCA3	0	0.2926
Frequency-MFCC-PCA4	0	0.4885
Frequency-MFCC-PCA5	0	0.2856
Frequency-MFCC-PCA6	0	0.2952
Frequency-Delta-PCA1	1	0.0001 *
Frequency-Delta-PCA2	0	0.1530
Frequency-Delta-PCA3	0	0.0579
Frequency-Delta-PCA4	0	0.1624
Frequency-Delta-PCA5	1	0.0369 *

**Table 4:** Classification results of vocal patterns of HC subjects and PD patients. N/A: Not applicable.

**Classification** **Metrics**	Methods				
	Bayesian Expert System [36]	Two-Stage Method [17]	KNN (K=7)	SVM	SSCL
Accuracy ± SD	0.752 ± 0.086	0.779 ± 0.08	0.806 ± 0.031	0.825 ± 0.03	0.838 ± 0.029
Recall ± SD	0.718 ± 0.132	0.765 ± 0.135	0.812 ± 0.044	0.8 ± 0.045	0.825 ± 0.042
Specificity ± SD	0.786 ± 0.135	0.792 ± 0.15	0.8 ± 0.045	0.85 ± 0.04	0.85 ± 0.04
Precision ± SD	0.785 ± 0.118	0.806 ± 0.115	0.802 ± 0.044	0.842 ± 0.042	0.846 ± 0.041
F-score ± SD	0.75 ± 0.024	0.785 ± 0.022	0.807 ± 0.012	0.821 ± 0.011	0.835 ± 0.011
MCC ± SD	0.505 ± 0.096	0.557 ± 0.089	0.613 ± 0.049	0.651 ± 0.046	0.675 ± 0.043
AUC ± SD	N/A	0.879 ± 0.067	0.855 ± 0.029	0.868 ± 0.043	0.939 ± 0.018
Kappa ± SD	N/A	N/A	0.613 ± 0.062	0.65 ± 0.06	0.675 ± 0.058

**Table 5:** Summary of the misclassified voice records in percentage and their corresponding subject group and gender information.

**Subject Group**	KNN		SVM		SSCL	
	Male	Female	Male	Female	Male	Female
HC	25.8%	25.8%	25%	17.9%	30.8%	15.4%
PD	38.7%	9.7%	46.4%	10.7%	53.8%	0%
Total	64.5%	35.5%	71.4%	28.6%	84.6%	15.4%
	100%		100%		100%	

## Data Availability

Not applicable.

## Abbreviations

The following abbreviations are used in this manuscript:

APQ	Amplitude Perturbation Quotient
AUC	Area Under the receiver operating characteristic Curve
DFA	Detrended Fluctuation Analysis
KNN	*K*-Nearest Neighbor
GNE	Glottal-to-Noise excitation Ratio
HC	Healthy controls
HNR	Harmonic-to-Noise Ratio
MCC	Matthews Correlation Coefficient
MFCC	Mel-Frequency Cepstral Coefficient
PCA	Principal Component Analysis
PD	Parkinson’s Disease
PPE	Pitch Period Entropy
ROC	Receiver Operating Characteristic curve
RPDE	Recurrence Period Density Entropy
SE	Standard Error
SSCL	Semi-Supervised Competitive Learning algorithm
SVM	Support Vector Machine
